# Management of Diabetes in Nursing Homes: Nurses' Perceptions on Diet and Meal Schedules

**DOI:** 10.1155/nrp/5542247

**Published:** 2025-10-15

**Authors:** Ana Domínguez-Navarro, Consuelo López-Fernández, Juan Manuel Picardo-García

**Affiliations:** ^1^Department of Nursing and Physiotherapy, Faculty of Nursing and Physiotherapy, University of Cádiz, Cádiz, Spain; ^2^University of Cádiz, Cádiz, Spain

**Keywords:** aged (MeSH), diet (MeSH), nurses (MeSH)

## Abstract

**Introduction:**

Diabetes affects more than 25% of elderly residents in nursing homes in Cádiz, Spain. Historically, therapeutic diets in these centers have included restrictions on calories, fats, and sugars, which may reduce food intake and increase the risk of malnutrition. In recent years, more flexible dietary approaches have emerged, aiming to improve both nutritional status and quality of life.

**Objective:**

To explore the perceptions and experiences of nurses in nursing homes regarding dietary management and meal schedules for residents with diabetes.

**Methods:**

A qualitative study was conducted with in-depth interviews of 18 nurses from nursing homes in Cádiz. Data were analyzed thematically.

**Results:**

Three major positions emerged: (1) Support for dietary flexibility, reported by 8 out of 18 nurses, who emphasized the positive effects of relaxed dietary restrictions on residents' quality of life and emotional well-being. (2) Preference for strict diabetic diets, supported by 4 nurses, highlighting the importance of sugar-free and low-carbohydrate alternatives for glycemic control. (3) Criticism of current institutional meal schedules, expressed by 6 nurses, which were seen as rigid, contributing to long fasting periods and increased risk of nocturnal hypoglycemia. Nurses also noted that these schedules prioritize staff convenience over resident-centered care.

**Conclusions:**

Dietary management and institutional meal schedules pose significant challenges to diabetes care in nursing homes. Incorporating greater dietary flexibility and adapting meal schedules to residents' individual needs may enhance both glycemic control and overall well-being.

## 1. Introduction

Diabetes mellitus is a prevalent and complex condition among older adults, particularly those residing in long-term care facilities. In Spain, nearly one in four adults over the age of 75 is diagnosed with diabetes [1, 2], with prevalence rates rising to 26.44% in institutionalized older populations in Cádiz [3]. Compared to community-dwelling individuals, nursing home residents with diabetes are often frailer, experience higher rates of cognitive impairment and functional decline, and are at increased risk for hypoglycemic events and other complications [4–6].

Effective management of diabetes in nursing homes is challenging due to the high clinical heterogeneity of residents, polypharmacy, and the interplay between chronic conditions, nutritional status, and care routines. Nutritional management plays a central role in diabetes care, yet traditional approaches have commonly involved restrictive therapeutic diets that limit calories, sugars, and fats. While such restrictions may aim to improve glycemic control, they can inadvertently lead to reduced food intake, unintentional weight loss, and malnutrition—particularly among older adults with diminished appetite, dysphagia, or cognitive decline [7, 8].

In response to these concerns, a shift toward more liberalized, resident-centered dietary practices has been observed in some long-term care settings. These approaches promote dietary flexibility by accommodating individual preferences, cultural habits, and quality-of-life considerations. Studies suggest that such flexibility can improve nutritional intake and psychological well-being without compromising glycemic outcomes [9]. However, the extent to which these strategies are implemented in institutional care—and the factors that enable or hinder them—remains unclear.

Meal timing and institutional scheduling also play a critical role in diabetes management. Rigid mealtimes, often organized around staff workflows rather than individual needs, can contribute to prolonged fasting periods, poor medication alignment, and nocturnal hypoglycemia [10]. Despite international guidelines emphasizing individualized care planning for older adults with diabetes [5], the operational structures of many nursing homes may limit the personalization of dietary routines.

Nurses are frontline providers in long-term care and hold a pivotal role in managing both the clinical and emotional dimensions of diabetes care. Their perspectives offer critical insights into the practical challenges and ethical tensions that arise when balancing therapeutic goals with residents' preferences and well-being. While some studies have focused on clinical outcomes or institutional practices, few have examined the lived experiences of nursing staff in implementing and negotiating dietary care strategies in these complex environments [11, 12].

This study addresses that gap by exploring the perceptions of nurses working in nursing homes regarding dietary management and institutional meal schedules for residents with diabetes. Understanding their experiences provides valuable direction for improving diabetes care in long-term care settings and advancing more person-centered, flexible, and effective dietary practices.

## 2. Methods

### 2.1. Study Design

This qualitative, phenomenological study was designed to explore the perceptions and experiences of nurses regarding dietary management and meal scheduling for diabetic residents in nursing homes. The phenomenological approach was selected for its capacity to capture the depth and complexity of nurses' lived experiences in managing diabetes within institutional care.

This qualitative study was designed and reported in accordance with the Consolidated Criteria for Reporting Qualitative Research (COREQ) tool, which provides a comprehensive framework to ensure transparency and quality in qualitative research. The assessment of compliance with the 32 criteria, organized into three domains, ensures that the methods employed and the results obtained adhere to internationally recognized standards [13]. A detailed evaluation of these criteria is included in Appendix A.

### 2.2. Eligibility Criteria

Participants were required to:

◦ Be nurses working in a nursing home in Cádiz, with responsibilities that include direct care for residents with diabetes.
◦ Have at least 6 months of work experience in a nursing home setting.

### 2.3. Sampling Methodology

Intentional sampling was employed to select participants who could provide rich, detailed data. This phase followed an initial quantitative study, and participants from that phase were invited to join this qualitative phase via a questionnaire.

### 2.4. Recruitment and Data Collection Process

Nurses who agreed to participate in the qualitative phase were contacted via email, which served as the primary means of communication. This approach facilitated effective coordination across the geographically dispersed province of Cádiz.

A total of 129 nurses were initially contacted. Of these, 63 responded positively and met the eligibility criteria—namely, working in nursing homes and having experience in diabetes care. From these 63, nurses were gradually invited to participate until data saturation was achieved. The 18th interview marked the point at which no new relevant insights emerged, concluding the recruitment process.

#### 2.4.1. Sampling Strategy

A purposive, criterion-based sampling approach was used to ensure diversity in participants' academic backgrounds, years of experience (both overall and within nursing homes), and workplace characteristics (e.g., public, private, or subsidized institutions; facilities with fewer or more than 100 residents).

#### 2.4.2. Final Sample

The final sample consisted of 18 nurses, whose sociodemographic characteristics are presented in Table 1. All other nurses who expressed interest but were not selected received a personalized email thanking them for their willingness to participate. The research team also committed to sharing the main findings with them as a gesture of appreciation and transparency.

Interviews were conducted in private settings to ensure confidentiality, with no third parties present. Each interview was conducted once and supported by field notes taken by the interviewer to complement the audio recordings during transcription. Interview durations ranged from 27 min to 1 h and 6 min, allowing participants to share their views freely. While all participants were offered the opportunity to review their transcripts, none requested to do so. Nevertheless, transcripts would have been shared with any participant who expressed interest, further ensuring transparency and accuracy in the data collection process.

### 2.5. Data Collection

In-depth, semistructured interviews were conducted via telephone between December 2023 and January 2024 to accommodate the broad geographic distribution of participants. The interviews, each lasting from 27 min to over an hour, were recorded with participants' consent. Field notes were also taken during the interviews to capture additional observations and contextual information. Only the interviewer and the participant were present during each interview to ensure privacy. The interviews followed a semistructured guide designed to elicit views on dietary flexibility, the necessity of strict diabetic diets, and institutional meal schedules.

### 2.6. Interviewer Background and Reflexivity

All interviews were conducted by the same female predoctoral nurse researcher, ensuring consistency throughout the data collection process. A professional relationship was established solely for the purposes of this study, and participants were informed about the interviewer's role and background to foster trust and transparency.

The interviewer's background in nursing and diabetes care supported a context-sensitive approach to data collection. It is worth noting that her background as a diabetes specialist could have subtly influenced the framing of questions or interpretation of responses. Throughout the interviews, she maintained a neutral stance, used open-ended and nonleading questions, and encouraged participants to express their views freely. Field notes were taken to support reflexive practice and enhance the credibility of the findings. Participants were also given the opportunity to review their transcripts, although none requested changes. This may reflect a high level of trust in the research process and interviewer or limited availability and competing responsibilities among participants working in time-constrained clinical settings.

These steps were taken to ensure transparency, rigor, and fidelity to participants' perspectives.

### 2.7. Data Analysis

Interview transcripts were analyzed through inductive content analysis using ATLAS.ti 9 software, with two researchers involved in coding to enhance the rigor and credibility of the findings. Themes were derived directly from the data, and the Delphi technique was employed to achieve consensus among coders, ensuring the consistency and relevance of the main themes, which included dietary flexibility, strict diet adherence, and meal schedule challenges.

A simplified coding tree outlining the main themes and subcategories is provided in Appendix B (Table A1) to enhance methodological transparency.

### 2.8. Consensus Building

Once the main codes were obtained, consensus and organization were sought using the Delphi technique. The Delphi technique was used with the following objectives: (1) to allow further reflection by participants, (2) to collectively organize the identified factors, (3) to explore divergences, and (4) to reach consensus on external and potentially modifiable factors influencing diabetes control.

Following the final analysis of the interviews, a checklist of factors was developed and paired with a five-point Likert scale (0 = strongly disagree, 5 = strongly agree). All 18 participants were invited via email to rate each item and provide comments, and all nurses responded. Consensus was defined a priori as a mean agreement score of ≥ 3.5. Based on this criterion, consensus was reached in the first round for the following items:

– “Diabetic flexibility” (4.82/5)
– “Meal schedules” (4.19/5)
– “Formality in diabetic diets” (4.69/5)

The use of a single round was justified by the fact that full consensus was reached immediately. This outcome may be partly explained by the participants' clinical experience and limited research background. Despite prior explanation of the Delphi process, participants tended to agree with the themes presented rather than offer dissent. While this ensured clarity and participation, it also represents a limitation, as the single round reduced the opportunity for iterative refinement or exploration of divergent views.

### 2.9. Ethical Considerations

The study received approval from the Ethics Committee of Research of Cadiz, Spain (PEIBA0652-N-23), ensuring all procedures were in line with the Declaration of Helsinki. All participants received detailed information about the study's objectives, procedures, and their rights, and each signed an informed consent form prior to participation.

To ensure confidentiality, all identifying information was removed during transcription. Participants were assigned alphanumeric codes (e.g., Nurse 8 became “N8”) to anonymize their responses, and no personal or institutional identifiers were included in the transcripts or reporting. Only the interviewer had access to the raw data, which was stored securely in password-protected files. These measures were implemented to safeguard participant identities and uphold strict confidentiality throughout the research process.

## 3. Results

Through in-depth interviews with 18 nurses from various nursing homes in the province of Cádiz, three overarching perspectives on diabetes-related dietary management emerged: (1) support for dietary flexibility, (2) preference for strict diabetic diets, and (3) concern over rigid institutional meal schedules. These perspectives reflect nurses' experiences balancing clinical guidelines with residents' well-being in long-term care settings.

### 3.1. Support for Dietary Flexibility

A significant number of nurses supported dietary flexibility—defined as the occasional relaxation of dietary restrictions to improve residents' quality of life. This perspective emphasizes a more holistic view of care, prioritizing emotional and cognitive well-being alongside glycemic control.

Several nurses described how allowing residents to enjoy familiar and comforting foods could foster positive emotional responses, particularly in older adults with cognitive decline or nearing end-of-life stages. This approach was often informed by professional experience and ethical considerations around dignity in aging.

A nurse with 17 years of experience states: *“Offering a chocolate to a diabetic person is not as bad as maintaining a strict diet, as this also helps the person's cognitive state.”* (N6). This reflects a broader belief that dietary leniency, when applied judiciously, can enhance quality of life without necessarily undermining clinical goals.

Another nurse illustrated the ethical and emotional dilemmas involved in restricting favorite foods for elderly residents, particularly those at the end of life: “*At 102 years old, if her nephew comes once a month and brings her cream-filled buns that she likes, are we not going to let her eat the cream-filled buns? So, she eats the cream-filled buns, enjoys her cream-filled buns, and we know that her blood sugar will rise, and we will have to lower it, that's it*!” (N14).

This quote highlights how nurses sometimes prioritize residents' emotional and social well-being over strict metabolic control, especially in the context of advanced age and limited life expectancy.

Of the 18 nurses interviewed, 8 agreed that allowing foods not recommended in strict diabetic diets can have cognitive and emotional benefits for residents, highlighting the importance of dietary flexibility as a strategy to improve quality of life.

This theme reflects a common view among participants (8 out of 18) that dietary flexibility can enhance emotional well-being and cognitive engagement in residents. Nurses emphasized ethical and person-centered motivations, particularly in end-of-life or dementia care. However, this perspective sometimes conflicted with institutional dietary protocols, leading to informal adaptations rather than formalized practices.

### 3.2. Preference for a Strict Diabetic Diet

In contrast, some nurses stressed the importance of strict adherence to diabetic diets, viewing such regimens as essential for maintaining glycemic control and preventing complications. This group emphasized the use of sugar-free and low-carbohydrate alternatives as effective strategies for managing diabetes within an institutional environment.

One participant stated, *“I start from the basis that sugary supplements are poison to me. They are poison. Because we get the glucose we need from the same foods, they have fructose, lactose, and so on, which are natural sugars.”* (N13).

In line with this conviction, several nurses praised breakfast and snack alternatives, such as sugar-free cookies and muffins, and low-carbohydrate lunch and dinner menus offered in some nursing homes. They argue that these are appropriate for daily consumption and provide an opportunity for effective glycemic control.

As one participant explained, *“We adapt the menu to the person who is diabetic (…) We try to reduce the number of sugars and carbohydrates;* for example*, diabetics have different bread from nondiabetics, whole grain bread, sugar-free, yogurts are fat-free for diabetics, besides, of course, sweeteners instead of sugar, and we reduce the number of high-sugar fruits,* for example*, bananas, and all that, it's adapted to diabetics.”* (N18).

Other participants negatively highlight the priority that some residential center staff place on residents eating, without attention to the characteristics of the foods consumed by elderly people with diabetes. As another nurse observed: *“The important thing here is that they have eaten, regardless of what and how.”* (N5). This comment underscores a disconnect between institutional feeding practices and individualized dietary care, suggesting that staff may focus more on completing tasks than on ensuring dietary alignment with clinical needs.

Nurses favoring strict dietary regimens (4 participants) prioritized glycemic control through structured, sugar-free, and low-carb diets. While their approach aligned with clinical norms, some tension emerged with colleagues' more flexible views and with practices that prioritized intake quantity over nutritional quality.

### 3.3. Oppositing to Existing Meal Schedule

It is common for meals in nursing homes to be organized as follows: breakfast between 9:00 and 10:30 a.m., lunch between 1:30 and 2:00 p.m., snack between 4:30 and 5:00 p.m., dinner between 7:30 and 8:30 p.m., and a late-night snack at 11:00 pm.

Nurses also identified current meal scheduling as a significant barrier to effective diabetes management, with existing structures typically grouping all meals within an 11-h period and leaving a fasting period of approximately 13 h. This extended fasting period was seen as increasing the risk of nocturnal hypoglycemia, particularly problematic for elderly residents who may already have disrupted sleep patterns and increased vulnerability to hypoglycemic events.

*“We have breakfast, mid-morning we give them a fluid intake, then lunch, snack, dinner, and a late-night snack to avoid long fasting hours.”* (N10).

Nurses identify that the intake of the late-night snack is compromised by factors such as insomnia medication administered at dinner and the risk of aspiration due to drowsiness. Additionally, the pharmacokinetics of oral medication is affected by meal schedules. Most oral medication is administered with meals, expecting 8 h between each meal, but in practice, it is less than 5 h between each intake. One nurse described the impact on medication timing:

*“If they are taking Metformin at breakfast, lunch, and dinner, they take it very close together, breakfast, lunch, and dinner, but then there are another twelve hours…, what should be three times in 24 h, is three times in 12 h and then another 12 h without taking anything. So, you end up adapting it based on glycemic controls, but adapting it to the schedule. As I said, the patient is adapted to the schedule rather than the schedule to the patient.”* (N4).

This quote illustrates how institutional routines often force clinical compromises, with schedules prioritized over patient-centered medication timing.

The prolonged fasting period between dinner and breakfast increases nocturnal hypoglycemia, which is difficult to identify as the resident is asleep. As one participant noted, late-night snacks felt like a short-term solution rather than addressing the underlying issue of inappropriate meal timing.

*“They go twelve hours without eating, unless we give them a ‘yogurt' (…), the base is the meal schedule. You are also waking up a patient who is already asleep because you gave them dinner pills at a quarter to eight in the evening, you are waking up a patient to give them a late-night snack, so they do not have hypoglycemia because of not having a good meal schedule.”* (N4).

This shows how staff resorted to stopgap solutions like late-night snacks rather than tackling the underlying problem of misaligned meal schedules.

Other participants shared concerns that the rigidity of institutional schedules was more oriented to staff efficiency than to meeting residents' individual needs.

*“The schedules, I think, are made more for the organization of work than for their well-being. So, it is a negative factor that also greatly affects glycemic control, from my point of view.”* (N8).

Of the 18 nurses interviewed, 6 highlighted problems with meal schedules. These perceptions indicate that adjusting meal schedules to better suit residents' needs could significantly improve glycemic control and overall well-being.

Six nurses raised concerns about rigid institutional meal schedules, particularly their contribution to prolonged fasting and misaligned medication timing. Participants described a misfit between residents' physiological needs and staff-driven routines, with late-night snacks seen as a workaround rather than a solution. This tension highlights broader organizational challenges in delivering individualized diabetes care.

## 4. Discussion

This study offers important insights into nurses' perceptions of dietary management and meal schedules for elderly residents with diabetes. Findings highlight the tension between strict glycemic control and efforts to improve quality of life, revealing divergent perspectives on dietary flexibility, adherence to therapeutic diets, and challenges arising from institutional meal scheduling.

Nurses advocating for dietary flexibility emphasized its benefits for residents' nutritional intake, emotional well-being, and autonomy. Their views align with a growing shift in long-term care toward more individualized and less restrictive dietary approaches. Dietary flexibility has been associated with improved food and beverage intake and reduced risk of malnutrition and unintentional weight loss in older adults [8]. Complementary strategies, such as fortified meals, have also shown benefits by increasing protein and energy intake among nursing home residents [14].

Nurses acknowledged that their training and clinical experience influence their openness to implementing more flexible dietary practices. In contrast, the preference for strict diabetic diets observed in some participants may reflect gaps in education or exposure to recent recommendations. Munshi et al. [15] noted that inconsistencies in knowledge, coupled with poor implementation of diabetes care protocols and lack of consensus on glycemic targets, contribute to suboptimal outcomes, including increased hospitalizations and reduced quality of life. This finding aligns with recent evidence on the importance of advanced clinical training and person-centered interventions in elderly care. Magi et al. [16] highlighted that tailored pain management strategies led by nurses significantly improved the comfort and safety of older hospitalized patients, reinforcing the need for skilled and specialized nursing staff in long-term care settings.

Despite the availability of guidelines for diabetes management in older adults, a review by Farrer et al. [17] highlighted that these documents rarely offer systematic approaches for achieving individualized nutrition in institutional settings. Nurses' perceptions in this study reinforce that existing menus may not adequately meet the glycemic and nutritional needs of residents. Because menus are central to the delivery of nutrition in nursing homes, persistent patterns of inadequate intake should prompt a review and possible revision of meal plans [18]. Tools such as the Quality Index for Nutrition in Nursing Homes (QUINN) can assist in assessing and improving the nutritional quality of menus [19]. Incorporating a variety of foods that reflect residents' preferences may improve both dietary adherence and glycemic control [20].

Concerns about rigid institutional meal schedules emerged as a consistent barrier to optimal diabetes management. Nurses noted that meal timing often compresses all daily meals into an 11 h window, followed by a prolonged fasting period of approximately 13 h. This pattern increases the risk of nocturnal hypoglycemia and may interfere with the pharmacokinetics of oral medications [10]. Moreover, several participants questioned whether meal schedules were structured more for operational convenience than resident well-being. These findings echo prior research showing that institutional cultures often prioritize staff routines over residents' individual needs [12]. Residents themselves report a desire for more traditional foods, a less monotonous diet, and the ability to eat at their own pace and at consistent times [21]. Similar concerns were identified in a Canadian study, where residents were given a snack at approximately 7 p.m. and no further food until breakfast the following day, creating a fasting period of 13.5 h [22].

To fully understand their implications, these results must also be interpreted in the context of broader structural and cultural factors that influence care delivery. Nurses' concerns about rigid schedules, lack of individualized dietary plans, and constraints in adapting care routines reflect deeply embedded institutional priorities that often favor efficiency over personalization. Such barriers are consistent with the structural constraints described in geriatric nursing, where staffing limitations, policy rigidity, and hierarchical decision-making restrict individualized care practices [23]. In practice, these systemic conditions mean that recommendations such as liberalizing diets or adjusting meal schedules, though valued by nurses, may be difficult to implement consistently. Limited staffing and high turnover reduce the time available for individualized support, while standardized food policies and cost-containment measures leave little room to adapt menus to residents' needs [22, 23]. Comparable structural barriers have also been reported in community-based settings. A qualitative study by Capitani et al. [24] revealed that home care nurses in rural Italy face professional isolation, lack of institutional support, and limited resources—challenges that strongly mirror the systemic issues described by participants in the current study.

Taken together, these systemic factors—limited budgets, standardized regulations, staff shortages, and hierarchical decision-making—illustrate why nurses' recommendations for dietary flexibility or adjusted meal schedules often remain aspirational. Without organizational reforms that address funding, staffing, and policy rigidity, person-centered dietary care is difficult to achieve, even when frontline providers recognize its benefits [22–24].

These are dynamic echoes of the principles of the culture change movement in long-term care, which promotes transforming traditional, hospital-like environments into resident-centered and relationally focused settings. Though the term “culture change” does not have a single universal definition, it encompasses models such as the Eden Alternative, the Green House model, and household/neighborhood care models. Across these frameworks, key features include (1) individualizing care; (2) creating homelike environments; (3) fostering close relationships between staff, residents, and families; (4) empowering frontline workers to respond to residents' needs in real time; and (5) engaging in continuous quality improvement. These principles directly address the issues raised by participants in this study. The rigidity of current meal schedules and lack of responsiveness to individual preferences represent institutional patterns that undermine resident autonomy and holistic care. By flattening traditional hierarchies and enabling staff to make bedside decisions, facilities may be better positioned to implement flexible dietary practices that support both glycemic control and residents' quality of life [25].

In this regard, many comprehensive care models have emphasized the central role of person-centered care, interdisciplinary team (IDT) assessments, and complex care management as key elements of high-quality care for older adults. According to McNabney et al. [23], person-centered care involves creating individualized care plans that empower residents to actively participate in decisions about their health and daily routines—an approach particularly crucial given the heterogeneity of the geriatric population. IDT assessments, while resource-intensive, provide valuable input from diverse healthcare professionals and are especially beneficial for frail or complex residents. Complex care management, often linked with care transitions, supports the continuity and coordination of care necessary to manage multifaceted clinical needs effectively [23].

Improving residents' quality of life requires organizational changes that minimize the negative effects of institutionalization, including limited autonomy and depersonalized care routines. Communication, staffing levels, and continuity of care are critical elements influencing residents' experiences. Prior studies have noted that high staff turnover, inadequate supervision, and a lack of individualized care planning are common challenges in nursing home environments [11, 26].

Taken together, the findings of this study support the call for more balanced and individualized care practices that reflect both the medical and emotional needs of elderly residents with diabetes. Promoting dietary flexibility, adapting meal schedules to individual needs, and addressing organizational barriers through culture change principles and team-based care approaches may contribute to improved glycemic outcomes and enhanced quality of life in institutional settings.

### 4.1. Limitations and Future Research

This study offers valuable insights, but some considerations should be noted. It focused on a specific geographic area (Cádiz), and while this allowed for in-depth exploration of the local context, future studies could examine similar themes across diverse settings to compare experiences and practices. Qualitative research does not aim to generalize findings. Its purpose lies in the exploration, understanding, and description of participants' experiences and life worlds, with the potential for theory to emerge from the data [27].

Although purposive sampling ensured diversity in participants' backgrounds and data saturation was achieved, this study focused exclusively on the perspectives of nurses. While this approach provided valuable insight into frontline clinical experiences, it did not include the views of residents with diabetes or their family members. This is a recognized limitation, as their perspectives would offer complementary understanding of how dietary routines and institutional practices are experienced. However, in the context of this study, nurses were the most accessible group and served as an appropriate starting point for examining this understudied area of care. Future research should build on these findings by incorporating the voices of residents and families to enrich the understanding of person-centered dietary management in nursing homes.

Additionally, the study may be subject to a volunteer bias, as participation was based on self-selection. It is possible that nurses who were more engaged, reflective, or experienced in diabetes care were more inclined to participate, potentially skewing the findings toward more informed or proactive perspectives. Although the Delphi round achieved full consensus, the lack of dissenting responses may also reflect participants' tendency to align with presented themes rather than challenge them. This reinforces the need to interpret consensus scores with caution.

Finally, the fact that all participants worked within the same regional policy framework provides a coherent picture of how systemic conditions shape practice in nursing homes. While the study does not aim for statistical generalization, the organizational challenges identified here are unlikely to be unique to Cádiz. Similar issues have been reported in long-term care facilities internationally [12, 22, 24], suggesting that these insights can contribute to broader discussions on dietary management and meal scheduling in other institutional contexts.

## 5. Conclusions

Dietary management and meal schedules in nursing homes present significant challenges for glycemic control in residents with diabetes. However, dietary flexibility and the adaptation of meal schedules to individual needs can offer opportunities to improve both the health and overall well-being of residents. This study highlights the importance of a balanced and personalized approach to dietary care and suggests the need for more flexible policies tailored to the preferences and needs of residents to promote healthy aging and better quality of life.

## 6. Practical Recommendations for Nursing Practice and Policy

Based on the findings, the following recommendations may improve diabetes care for elderly nursing home residents:

• Implement Flexible Dietary Policies:
  Based on the theme of support for dietary flexibility expressed by 8 out of 18 nurses, policies allowing selective inclusion of preferred foods may improve satisfaction and mitigate cognitive decline. As one nurse emphasized, allowing occasional treats like chocolate or cream-filled buns can support emotional and cognitive well-being (N6, N14).
• Revise Meal Schedules:
  This recommendation addresses the theme of opposition to existing meal schedules, expressed by 6 nurses. Participants described how compressed mealtimes and prolonged fasting (e.g., 13 h overnight) contribute to nocturnal hypoglycemia and medication misalignment (N4, N10). Revising mealtimes to better match residents' needs could improve both safety and glycemic stability.
• Pilot Flexible Policy Programs:
  To operationalize the findings, facilities may test new scheduling and dietary protocols through pilot programs. This is grounded in nurses' observations that institutional routines often reflect staff convenience rather than resident-centered care (N8). Pilots would allow for real-world testing of flexibility, aligning care practices more closely with the values and concerns expressed by nursing staff.

## Figures and Tables

**Table 1:** Sociodemographic characteristics of participants.

Sociodemographic characteristics		Percentage/mean
Gender	Women	88.9%
	Men	11.1%

Age (mean)		37.8 years old

Education	Specialist in geriatric	16.67%
	Master's degree	16.67%
	PhD	0%

Courses	Yes	44.4%
	No	55.6

Diabetes courses	Yes	38.89%
	No	61.11%

Years of professional experience (mean)		13.4

Years of professional experience in nursing homes (mean)		10.6

Years of professional experience in diabetes care (mean)		12.7

Diabetes in relatives	I have diabetes	0%
	A family member has diabetes	38.9%
	A friend has diabetes	5.6%
	No acquaintances with diabetes	50.0%
	Gestational diabetes	5.6%
	A family member and a friend have diabetes	0.0%

Interview duration (mean, min)		17.93

**Table 2:** Coding tree of major themes and subcategories.

Dietary flexibility	Emotional well-being
	Cognitive support
	End-of-life considerations

Strict diabetic diet adherence	Use of sugar-free foods
	Diabetic menu
	Nurse preference

Institutional meal schedules	Fasting duration
	Staff-centered routines

## Data Availability

The data generated and analyzed during the current study are not publicly available due to ethical and confidentiality restrictions. However, it is available from the corresponding author on reasonable request.

## Appendix A: COREQ: A 32-Item Checklist for Interviews and Focus Groups

To ensure transparency and rigor, this study adheres to the COREQ checklist, a 32-item guide for comprehensive reporting in qualitative studies that utilize interviews and focus groups. By aligning with COREQ, we provide detailed insights into the perspectives of nurses on managing diabetes in nursing homes, from the background and reflexivity of the research team to the nuances of participant recruitment, data collection, and thematic analysis. This approach allows us to present the diverse views of nurses on dietary flexibility, strict dietary adherence, and meal scheduling challenges in ways that are both systematic and relatable. Through COREQ, we aim to offer a thorough and accessible contribution, inviting readers to apply these insights to enhance diabetes management and resident-centered care in similar institutional settings [13].

Domain 1: Research Team and Reflexivity.

1. Interviewer: Interviews were conducted by a female predoctoral nurse researcher.
2. Credentials: Bachelor's degree in Nursing, Master's degree in Health Sciences, and current PhD candidate.
3. Occupation: PhD student and nursing researcher at the University of Cádiz during the study.
4. Gender: Female.
5. Experience and Training: The interviewer had formal training in qualitative research methods and previous experience conducting interviews in healthcare settings.
6. Relationship Established: No prior relationship with participants. A professional rapport was established during initial contact for the study.
7. Participant Knowledge of the Interviewer: Participants were informed about the interviewer's academic background, clinical expertise in diabetes care, and study objectives.
8. Interviewer Characteristics: The interviewer's nursing background and experience in diabetes management supported sensitivity during interviews and minimization of potential biases.

Domain 2: Study Design.

9. Methodological Orientation: A phenomenological approach was used to explore participants' lived experiences regarding diabetes care in nursing homes.
10. Sampling: Purposive sampling strategy was employed, focusing on nurses with direct experience managing residents with diabetes.
11. Method of Approach: Initial contact was made via email invitations, followed by email and telephone coordination.
12. Sample Size: 18 nurses participated.
13. Nonparticipation: Of 129 nurses contacted, 63 responded positively. Sampling ceased once data saturation was reached at 18 interviews.
14. Setting of Data Collection: Interviews were conducted individually by telephone to accommodate participants across the Cádiz province.
15. Presence of Nonparticipants: Only the interviewer and the participant were present during each interview.
16. Description of Sample: Participants were nurses working in public, private, or subsidized nursing homes, with diverse backgrounds in geriatrics, chronic care, and diabetes management.
17. Interview Guide: A semistructured interview guide was developed based on a literature review and expert consultation. It was pilot-tested internally before use.
18. Repeat Interviews: No repeat interviews were conducted.
19. Audio/Visual Recording: All interviews were audio-recorded with participants' prior consent.
20. Field Notes: Field notes were made during and after each interview to capture contextual and nonverbal observations.
21. Duration: Interviews lasted between 27 min and 1 h 6 min (mean 47 min).
22. Data Saturation: Data saturation was determined when no new themes or concepts emerged after the 18th interview.
23. Transcripts Returned: Participants were offered the opportunity to review and correct their transcripts; none requested modifications.

Domain 3: Analysis and Findings.

24. Number of Data Coders: Two researchers independently coded the data and discussed discrepancies to enhance reliability.
25. Description of the Coding Tree: Codes were organized into themes based on emerging patterns; a coding tree diagram was developed internally but not included in the article.
26. Derivation of Themes: Themes were inductively derived directly from the data during content analysis.
27. Software: ATLAS.ti version 9.0 software was used for coding and thematic organization.
28. Participant Checking: Participants were not involved in checking the final analysis but could review their interview transcripts if they wished.
29. Quotations Presented: Direct quotations from participants are included to illustrate major themes, identified by participant codes (e.g., N4 and N8).
30. Data and Findings Consistent: There is a clear consistency between the data collected and the themes reported.
31. Clarity of Major Themes: Major themes (support for dietary flexibility, strict dietary adherence, and criticism of institutional meal schedules) are clearly presented.
32. Clarity of Minor Themes: Minor variations in perspectives were also described to reflect the range of views across participants.
